# Diagnostic testing in psychiatry: insights and examples from a Bayesian perspective

**DOI:** 10.1177/10398562241300887

**Published:** 2024-11-21

**Authors:** Paul A Maguire, Jeffrey CL Looi

**Affiliations:** Academic Unit of Psychiatry and Addiction Medicine, School of Medicine and Psychology, 2219 The Australian National University, Canberra Hospital, Canberra, ACT, Australia; Consortium of Australian-Academic Psychiatrists for Independent Policy and Research Analysis (CAPIPRA), Canberra, ACT, Australia; Academic Unit of Psychiatry and Addiction Medicine, School of Medicine and Psychology, 2219 The Australian National University, Canberra Hospital, Canberra, ACT, Australia; Consortium of Australian-Academic Psychiatrists for Independent Policy and Research Analysis(CAPIPRA), Canberra, ACT, Australia

**Keywords:** Bayesian, posterior probability, prior probability, anti-NMDAR encephalitis, clinical utility

## Abstract

**Objective:**

To demonstrate the application of Bayes’ theorem to diagnostic testing in clinical settings, especially with respect to rare diseases, enhancing an understanding of pre-test probability and its implications.

**Conclusion:**

Bayes’ theorem enables the revision of the conditional probabilities of an event occurring when new information is acquired. It demonstrates that when the prevalence of a disease is very low, there are a high number of false positives, thereby reducing the clinical utility and cost benefit profile of the diagnostic test, even in the presence of relatively high sensitivities and specificities of the chosen test.

‘Not everything is as it seems, and not everything that seems is’. (Jose Saramago)

Bayes’ theorem has explanatory power for why there are significant limitations in diagnostic testing of rare diseases, due to the high number of false positives. It also has relevance by encouraging clinicians to find ways of increasing the Bayesian prior probability to enhance the clinical utility of testing, by improving the chances of diagnostic accuracy.

## Brief history

Presbyterian minister, mathematician, and philosopher, Reverend Thomas Bayes (1701–1761), formulated his famous ideas on conditional probability in the mid-18^th^ century (Table 1).^1^ However, he did not consider them worth publishing or presenting to the Royal Society (of which he was a member). Following his death, Bayes’ relatives gave his papers to a friend, Richard Price (also a mathematician and philosopher), who subsequently published Bayes’ paper (in 1763), titled, ‘An essay towards solving a problem in the Doctrine of Chances’.^2^ Pierre-Simon Laplace independently conceived similar concepts of using new evidence to update probabilities and developed corresponding useful mathematical representations. Subsequently, Bayes’ theorem has received high praise and recognition, including the assertion that Bayes’ theorem is to the theory of probability what the Pythagorean theory is to geometry.^2^

**Table 1.** Thomas Bayes’ thought experiment

Bayes described a thought experiment of a man sitting with his back to a perfectly flat and square table. A person throws a ball onto this table, followed by further balls one after the other. Each time the person throwing the balls tells the seated individual where the most recently thrown ball lies in relation to the first ball. Bayes pointed out that the seated man can form an increasingly better estimation of where the original ball lies on the table, through this repeatedly updated information.

## What is Bayes’ theorem?

Bayes’ theorem enables revision of an existing prediction or estimated likelihood of an event occurring, when new evidence or additional relevant information becomes available.^3^ Bayes’ theorem has been widely used, including in science, medicine, and economics.^3,4^

Bayes’ formula states that the probability of an event A, given an event B (the new information), equals the probability of A times the probability of event B given A, divided by the probability of B. This can be represented mathematically as:

$$P\left(A|B\right)=\frac{P\left(A\right)\times P\left(B|A\right)}{P\left(B\right)}$$

‘Prior probability’, P(A), is the probability of a given event occurring before the new evidence or additional information is known (e.g. the prevalence of a disease). It represents the best rational estimation of the probability of a given outcome based on the existing knowledge at that time.

‘Posterior probability’, P(A|B), the probability of event A given event B, is the updated or revised probability of the specific outcome after taking into consideration the new information which has become available (the probability of having a disease after a diagnostic test shows a positive result).

P(B) is the ‘unconditional probability’ of event B occurring, and P(B|A) is the probability of event B occurring given that event A has occurred (e.g. the sensitivity of a diagnostic test).

## Usefulness of Bayes’ theorem in medical decision-making and disease testing

Bayes’ theorem can be helpfully employed in disease diagnosis. Consider a rare disease with a prevalence of 0.1% (one in a thousand) and a very accurate test for this disease which has a 99% sensitivity and 99% specificity. A patient may ask their clinician what is the likelihood of having the disease if they test positive. There are four possible outcomes: they test positive and have the disease, they test positive and don’t have the disease, they test negative and have the disease, or finally, they test negative and don’t have the disease. Table 2 indicates these outcomes if a hypothetical sample of 100,000 people were tested.

**Table 2.** Disease probability for hypothetical sample (100,000 people tested)

	Disease present (D^+^)	Disease absent (D^**−**^)	
Test is positive (T^+^)	99	999	1098
Test is negative (T^−^)	1	98,901	98,902
	100	99,900	100,000

Prevalence of disease = 0.1%.

Bayes’ formula can be used to calculate the probability of having the disease given a positive test result:

$$P\left(D^{+}|T^{+}\right)=\frac{P\left(D^{+}\right)\times P\left(T^{+}|D^{+}\right)}{P\left(T^{+}\right)}$$

where *P(D*^*+*^*|T*^*+*^*)* is the probability of having the disease if the test is positive;*P(D*^*+*^*)* is the prevalence of the disease (0.001);*P(T*^*+*^*|D*^*+*^*)* is the sensitivity of the test (0.99), that is, the probability that the test is positive if the disease if really present;*P(T*^*+*^*)* is the probability that the diagnostic test is positive.

The law of total probability was used to calculate the probability of the test yielding a positive result, that is, P(T^+^) includes both the true positives (99) and the false positives (999) totalling 1098 positives. Therefore, P(T^+^) is given by the equation:

$$P\left(T^{+}\right)=P\left(D^{+}\right)\times P\left(T^{+}|D^{+}\right)+P\left(D^{-}\right)\times P\left(T^{+}|D^{-}\right)=0.001\;x\;0.99+0.999\;x\;0.01=0.01098$$

Therefore, using Bayes’ formula, the probability of having the disease if the test is positive is

$$P\left(D^{+}|T^{+}\right)=\frac{P\left(D^{+}\right)\times P\left(T^{+}|D^{+}\right)}{P\left(T^{+}\right)}=\frac{0.001\times 0.99}{0.01098}=0.09$$

that is, there is only a 9% chance of having the disease.

Given that the test is highly accurate, this result seems counterintuitive. However, it makes more sense when we consider that because the disease is rare, the number of false positives markedly outnumbers the number of true positives, for example, in 1000 people, there will be only one person with the disease (a true positive), whereas there will be ten people with a false positive. One positive out of a total of 11 positives (i.e. 1/11) is 9%.

Interestingly, if the test is repeated and found to be positive a second time, the chance now that the individual has the disease increases to 91%. This is because further new information is added to the Bayesian equation, that is, the prior probability now is 9% (as shown above) rather than 0.1% (because the first test was positive).

$$P\left(D^{+}|T^{+}\right)=\frac{0.09\times 0.99}{\left(0.09\times 0.99\right)+\left(0.91\times 0.01\right)}=0.91=91\%$$

## Anti-NMDAR encephalitis as an example of a rare disease that causes psychiatric symptoms

Characterised in 2007 by Professor Josep Dalmau, anti-NMDAR encephalitis is an autoimmune disorder where NMDA-type glutamate receptors in the brain are bound to and blocked by antibodies (Table 3).^5^ Although the precise incidence and prevalence are unknown, the estimated incidence is 1.5 per million (population at risk) per year and the estimated prevalence is 0.6/100,000 (six cases per million people).^5,6^

**Table 3.** Summary of clinical features of anti-NMDAR encephalitis^5^

• Approximately 80% of cases occur in females, and 50% of these (for patients >18 years old) have an underlying tumour, mostly an ovarian teratoma
• In male patients, only about 5% have an identifiable tumour
• About 70% of patients experience a viral-like prodrome including lethargy, headache, respiratory symptoms, nausea, diarrhoea, or fever, lasting on average 5–7 days (no longer than 2 weeks)
• Psychiatric features (which follow prodrome) include delusions, especially persecutory, auditory, and/or visual hallucinations, agitation, changes in thought form such as perseveration, and generally disorganised thinking and behaviours; insomnia and cognitive deficits (including short-term memory difficulties and attentive deficits) are also common
• Neurological symptoms (which follow psychiatric symptoms) include abnormal movements such as orofacial dyskinesias, dystonic posturing, choreiform or athetotic movements, opisthotonos, and oculogyric crisis; seizures occur in about 70% of patients; autonomic instability may occur in this phase, especially tachycardia, hypertension, hyperthermia, and hypoventilation
• Recovery phase may take months to years, with prolonged deficits such as executive dysfunction and sleeping abnormalities
• About 75% of patients make a full recovery or have only mild residual deficits, and 25% remain severely disabled or die (4%)

NMDAR = N-methyl D-aspartate receptor.

Investigations include blood and cerebrospinal fluid (CSF) testing, magnetic resonance imaging (MRI) brain scan, electroencephalogram (EEG), and relevant imaging such as a pelvic ultrasound to exclude ovarian pathology (Table 4).^6^ Treatment is predominantly immunotherapy and if clinically indicated (e.g. for ovarian teratoma), surgery (Table 5).^6^

**Table 4.** Investigations for suspected anti-NMDAR encephalitis

Blood
• Anti-NMDAR antibodies
CSF
• Anti-NMDAR antibodies
• Moderate lymphocytic pleocytosis
• Oligoclonal bands (60% cases)
• Protein may be normal or elevated
MRI scan brain
• Abnormal in 50% cases – hyperintensities (cortical or subcortical regions)
EEG
• Usually abnormal – slowing
Imaging to exclude tumour
• Pelvic MRI scan or ultrasound for ovarian pathology
• Ultrasound scrotum to exclude testicular pathology

NMDAR = N-methyl D-aspartate receptor.

**Table 5.** Treatment of anti-NMDAR encephalitis^6^

Immunotherapy
First line
• Corticosteroids
# Intravenous methylprednisolone
# Oral prednisone
• IV Ig
• Plasma exchange
Second line
• Rituximab
• Cyclophosphamide
• Tocilizumab
• Bortezomib
• Mycophenolate mofetil
• Azathioprine
Surgery (if clinically indicated)
• Excision of ovarian teratoma
• Excision of testicular tumour

Ig = immunoglobulin; NMDAR = N-methyl D-aspartate receptor.

## Application of Bayes’ theorem to the serological diagnosis of anti-NMDAR encephalitis

The principles of Bayes’ theorem outlined above may be applied to serological antibody tests used in the diagnosis of anti-NMDAR encephalitis. Using cell-based assays (CBAs) and immunohistochemistry (IHC), estimates of the sensitivity of serological tests for diagnosis of anti-NMDAR encephalitis vary between 68% and 86%.^7,8^ In terms of specificity, a study has revealed a false positive rate of 23.2%, with anti-NMDAR antibodies occurring in patients with ultimately alternative diagnoses.^6^ Dahm et al. found about 10% false positivity for serum NMDAR antibodies in both healthy controls (*n* = 1703) as well as in patients with neuropsychiatric disorders including schizophrenia, mood disorder, cerebrovascular accident (CVA), Parkinson’s disease, amyotrophic lateral sclerosis, and personality disorders (*n* = 2533).^9^

Using the most favourable sensitivity and specificity estimates (i.e. 86% and 90%, respectively), Bayes’ formula regarding the likelihood of a patient (from the general population) having anti-NMDAR encephalitis if their serum antibody test is positive is given by:

$$P\left(D^{+}|T^{+}\right)=\frac{0.000006\times 0.86}{\left(0.000006\times 0.86\right)+\left(0.999994\times 0.1\right)}=0.0051\%$$

Even if the test was repeated and the result was again positive, the probability would still be very low (0.045%), using the updated prior probability (i.e. 0.0051% rather than 6/million).

## First episode psychosis (FEP) and serum anti-NMDAR antibodies

At the time of first onset of symptoms, it is often difficult to distinguish anti-NMDAR encephalitis from primary psychiatric conditions, especially schizophrenia. Clinical features such as persecutory delusions, agitation, perceptual disturbance, and disorganised thought form and/or behaviour are common in both conditions. However, early recognition of anti-NMDAR encephalitis and prompt treatment with immunotherapy is important as it can result in significant improvements in clinical outcomes (including long-term cognition).^10,11^

The estimated prevalence of anti-NMDAR encephalitis in patients presenting with FEP has been found to be about 1%.^12^ If this figure is used as the prior probability, the Bayesian formula now gives a result of 8% for the positive predictive value (PPV) of serum anti-NMDA antibodies testing. As described above, this PPV is low due to the high number of false positives related to the low prevalence of the disorder, even in these specific FEP cohorts. In a study of 112 patients with FEP, Kelleher et al. found an anti-NMDAR antibody seroprevalence of 3.5% (four cases).^12^ However, only one of these four patients had encephalitis. The other three were ultimately diagnosed with mood disorder exhibiting psychotic features, had normal EEGs and CSF analysis, and responded to standard psychiatric treatment. None of these three were found to have neurological abnormalities at 3-year follow-up.

The Royal Australian and New Zealand College of Psychiatry (RANZCP) clinical practice guidelines (from 2016) recommend screening of all patients with first episode psychosis with serum anti-NMDAR antibodies.^13^ However, *Bayesian statistics indicate a low yield with this approach*. Consequently, the RANZCP clinical practice guidelines for screening of all patients with FEP with serum anti-NMDAR antibodies^13^ are not supported on this analysis. Serum testing is associated with significant costs in the presence of a relatively low yield. Given the relevance and importance of the characteristic clinical features of patients presenting with anti-NMDAR encephalitis, researchers have developed specific clinical screening criteria to try to identify patients with first onset psychosis who are more likely to have this condition.

Scott et al.’s ‘screening recommended’ and ‘screening not recommended’ criteria (Table 6) can be used to determine who should undergo serological testing for anti-NMDAR antibodies, enabling selection of those with suitable pre-test probabilities (higher Bayesian prior probabilities).^14–17^ ‘Screening recommended’ is applied to patients with rapid onset of severe psychosis with additional symptom profiles such as neurological symptoms or marked cognitive deficit, which are typical for anti-NMDAR encephalitis but atypical for a schizophrenia or other primary psychiatric disorder. These screening criteria have been validated by Warren et al. using patients with confirmed anti-NMDAR encephalitis and controls, yielding a sensitivity of 97.3% and a specificity of 85.4%.^14^ Alternative screening criteria include Herken and Pruss ‘yellow flags’ criteria, which have also been validated.^14,18^

**Table 6.** Scott’s screening criteria^14^

Indications for anti-neuronal antibody (including anti-NMDAR antibodies) testing
*Rapid onset* of first episode of psychosis
PLUS one (or more) of:
• Neurological features (headaches, seizures, abnormal involuntary movements, or EPSE)
• Severe cognitive impairment
• Catatonia or intermittent agitation and excitation
• Antipsychotic hypersensitivity
• Need for ECT due to illness severity
Screening with anti-neuronal antibody (including NMDAR) testing NOT indicated
• Long prodrome of psychosis
• Duration of untreated psychosis > 4 weeks without requiring hospitalisation
• Intact cognition and language function
• Absence of neurological abnormalities

EPSE = extrapyramidal side effects; NMDAR = N-methyl D-aspartate receptor.

Singer et al., who have also identified universal antibody testing of all FEP patients as problematic, have listed the following clinical features as suggestive of anti-NMDAR encephalitis and have asserted that restricting serum NMDAR antibody testing would reduce the risk of false positives: young age, being female, no previous history of psychiatric illness, infectious prodrome, rapid onset, negative and positive symptoms at index presentation, motor abnormalities, reduced consciousness, seizure, sleep changes, speech dysfunction, autonomic dysfunction, and intolerance to antipsychotics.^16^

In addition to serum testing for relevant antibodies, patients suspected of having anti-NMDAR encephalitis may also be investigated with CSF analysis. There are considerably higher sensitivities and specificities for CSF antibodies in anti-NMDAR encephalitis, which improve diagnostic accuracy. Bastiaansen et al. found sensitivities of 93.0%–99.9% for both immunohistochemical and cell-based assay techniques, and specificities of 99.2%–99.8% for CBA and 99.7%–100% for IHC.^8^ In addition to presence of relevant antibodies, there may be other CSF abnormalities in patients with anti-NMDAR encephalitis. These include lymphocytic pleocytosis, elevated protein, and oligoclonal bands. However, a lumbar puncture is an invasive test and should not be performed without sufficient evidence for its clinical utility.^8^ There are logistic challenges (and added cost) associated with performing a lumbar puncture on all patients presenting with first onset psychosis (especially those with severe behavioural disturbance). This may place an unjustified burden and risk on both patients and medical/psychiatric staff.

Therefore, in people presenting with FEP there are competing tensions between, on one hand, not missing a diagnosis of anti-NMDAR encephalitis, a serious and debilitating but treatable disease, and, on the other hand, the cost and relatively low yield of serum antibody testing. Implementation of the appropriate specific treatment, immunotherapy, relies on an accurate diagnosis.

A limitation in this discussion of the application of Bayes’ theorem to anti-NMDAR encephalitis is the uncertainty of its true incidence and prevalence. In real-world clinical settings, many patients with first onset psychosis are not tested for anti-NMDAR antibodies and therefore may be missed diagnoses. Despite this, the best expert estimations suggest it is rare.

## Conclusion

Bayes’ theorem provides a mathematical and conceptual framework to assist in the assessment of the likelihood that a person has a disease, given the result of a chosen diagnostic test. It has value in the estimation of the cost benefit profile of performing a given test. By highlighting Bayesian reasoning, this paper emphasises and illustrates that even in the presence of relatively high sensitivities and specificities, the risk of having a disease is counterintuitively lower than expected if the disease is rare (low prior probability), due to the substantial number of false positives. Bayes’ theorem enables an update of the conditional probability of an event occurring when new information is acquired.

## Supplemental Material

Supplemental Material - Diagnostic testing in psychiatry: Insights and examples from a Bayesian perspective Supplemental Material for Diagnostic testing in psychiatry: Insights and examples from a Bayesian perspective by Paul A Maguire and Jeffrey CL Looi in Australasian Psychiatry
