# Supplementary material for: Diagnostic testing in psychiatry: insights and examples from a Bayesian perspective
Source: Australas Psychiatry. 2024 Nov 21;33(1):162–7. doi: 10.1177/10398562241300887 (PMC11804144; doi:10.1177/10398562241300887)
Supplement: Supplemental Material - Diagnostic testing in psychiatry: Insights and examples from a Bayesian perspective [file sj-pdf-1-apy-10.1177_10398562241300887.pdf]

Appendix

Cell-based assays and immunohistochemistry are both important laboratory techniques, that can be used in the screening of patients for anti-NMDAR encephalitis. Cell-based assays rely on the functional study of transfected human cells, most commonly using indirect immunofluorescence, whereas immunohistochemistry focuses on identifying specific staining reactivity in rat brain tissue exposed to the patient's serum or CSF sample.[1]

Cell-based assay

Human embryonic kidney (HEK)-239 cells are genetically engineered (using plasmids) to express NMDA receptors on their cell membranes. They are transfected with NMDA receptor subunits, typically NR1.[1] The patient's serum or CSF specimen is applied to these transfected cells where binding will occur if anti-NMDAR antibodies are present in the patient's sample. After this incubation with the patient's specimen, a secondary antibody, which has been conjugated to a fluorescent dye, is introduced, which will recognise and bind to (primary) anti-NMDAR antibodies attached to the transfected cells. These secondary antibodies are then detected on the cell surface using a fluorescence microscope. Commercially available kits are widely used. Most cell-based assays are fixed but some use unfixed live HEK cells.

Immunohistochemistry

Adults rats are injected with a fragment of NMDA receptor (or a similar protein) together with adjuvant agents (which boosts the immune response) to induce anti-NMDAR encephalitis. Passive transfer of human antibodies is sometimes also used for this purpose. These rats are subsequently anaesthetised and decapitated, followed by extraction of their brain tissue which is washed in phosphate-buffered saline, snap-frozen, or embedded in paraffin, and sectioned.[1] These sections are mounted on slides and used to detect anti-NDMAR antibodies by examining for reactivity in the form of a characteristic staining pattern. Cultures of dissociated rat hippocampal neurons can also be used in immunohistochemistry.[2] McCracken et al., when examining 290 serum samples detected anti-NMDAR antibodies in forty-one of these, but identified an additional seven (12% increase) when brain tissue immunohistochemistry was also used.[1] Bastiaansen et al. found sensitivities of 68% (range 55-78%) for CBA compared with 73% (range 61 – 83%) for immunohistochemistry when examining 126 patients with anti-NMDAR encephatilitis.[3]

Compared with immunohistochemistry, cell-based assays are cheaper, faster and more efficient and can be automated. Immunohistochemistry, although achieving better diagnostic sensitivity, is more labour-intensive and time-consuming as it involves tissue preparation, sectioning and staining, and more extensive microscopic examination.

References

1. McCracken L, Zhang J, Greene M, et al. Improving the antibody-based evaluation of autoimmune encephalitis. *Neurol Neuroimmunol Neuroinflamm* 2017; 4(6): e404.

2. Gresa-Arribas N, Titulaer MJ, Torrents A, et al. Diagnosis and significance of antibody titers in anti-NMDA receptor encephalitis, a retrospective study. *Lancet Neurol* 2014; 13: 167.

3. Bastiaansen AE, de Bruijn MA, Schuller SL, et al. Anti-NMDAR encephalitis in the Netherlands, focusing on late-onset patients and antibody test accuracy. *Neurology: Neuroimmunology & Neuroinflammation* 2021; 9: e1127.
